# Body Condition and Breeding of Urban Red Squirrels: Comparison of Two Populations Affected by Different Levels of Urbanization

**DOI:** 10.3390/ani12233246

**Published:** 2022-11-23

**Authors:** Agata Beliniak, Jakub Gryz, Daniel Klich, Karolina Jasińska, Dagny Krauze-Gryz

**Affiliations:** 1 Department of Forest Zoology and Wildlife Management, Warsaw University of Life Sciences, Nowoursynowska 159, 02-776 Warsaw, Poland; 2 Department of Forest Ecology, Forest Research Institute, Sękocin Stary, Braci Leśnej 3, 05-090 Raszyn, Poland; 3 Department of Animal Genetics and Conservation, Warsaw University of Life Sciences, Ciszewskiego 8, 02-786 Warsaw, Poland

**Keywords:** body mass, live-trapping, seasonal changes, breeding activity, *Sciurus vulgaris*, urban mammals

## Abstract

**Simple Summary:**

The red squirrel is among the mammals that have adjusted well to urban habitats. We compared two populations inhabiting Warsaw: in a park (with plentiful supplemental feeding) and in a forest (with no feeding and restricted visitor access). In previous studies, we showed that squirrels in the park differed from forest squirrels in space use, food choice, activity patterns and interactions with people. Now, we predicted that the supplemental feeding of park squirrels would result in their higher body mass, better body condition, a higher share of breeding females and extended breeding season. Contrary to our assumptions, forest squirrels were heavier and had better body conditions. Yet, in both populations, better body conditions increased chances for breeding. There were more breeding females and more young squirrels in the park. Squirrels from both areas bred mostly in spring but also in winter. The lower body mass/condition of park squirrels may have been due to competition in a high-density population, or may suggest that with year-round access to food, accumulating fat was not crucial. The extended breeding season may have been thanks to good feeding conditions and/or mild winters in the city. Again, we showed the high plasticity of red squirrels living in human-transformed habitats.

**Abstract:**

The red squirrel is among the mammals that have adjusted well to urban habitats. Here, we focused on the two populations inhabiting Warsaw: in a park (with year-round supplemental feeding) and in an urban forest. We hypothesised that park squirrels would have higher body mass (and better body condition), being more stable over the year, and would have a higher breeding rate (i.e., the share of breeding females). Contrary to our hypothesis, forest squirrels were heavier and had better body condition than park squirrels. The body masses of squirrels from both areas were quite stable (with the highest values obtained in spring). Females in better body conditions were more likely to breed. More breeding females and sub-adults were trapped in the park. Regardless of the study site, the highest share of breeding females was in spring, but they also bred in winter and in the remaining seasons. The lower body mass/condition of park squirrels may be possibly explained by high intraspecific competition, or by stable food (and thermal as typical for the city) conditions, in which accumulating fat was not crucial. Mild winter conditions may have also enabled squirrels to breed early. This study showed the high plasticity of red squirrels living in human-transformed habitats.

## 1. Introduction

Human populations have become increasingly urbanised and, as an effect, cites and their infrastructures are rapidly expanding [1]. These highly modified landscapes, however, may offer novel habitats for wildlife [2,3,4]. Anthropogenic changes in landscape can impose rapid evolutionary change [5]. Urban habitats differ from natural environments by their altered food availability, acoustic and light pollution, thermal conditions, or different sources and rates of mortality [4,6,7]. Nevertheless, many animal species adjust to urban conditions (e.g., [2,3,8,9,10,11]) by changing their diet preference, home range size, or behaviour [6].

The high availability of food in urban environments is thought to be among the reasons why many species of animals are thriving in cities (review in: [12]). Predictable anthropogenic food subsidies affect the body conditions and breeding parameters of individuals [13]. The effect of anthropogenic food sources may vary; urban vertebrates can benefit from increased food access and be in good body condition. Alternately, high food predictability can result in reduced body mass thanks to the limited need to accumulate body reserves. Finally, urban diet may lead to poor body condition in urban individuals due to low food quality (review in: [14]). In general, increased body mass and fertility are observed, yet this effect may be sex-dependent [13]. For example, male silver gulls (*Larus novaehollandiae* Stephens, 1826) from urban environments with access to an anthropogenic diet were heavier and had better body condition than non-urban male gulls, but no differences were detected between females [15]. On the other hand, sparrows (*Passer domesticus* L., 1758) in more urbanised habitats had reduced body size and body mass compared to their rural conspecifics but with no differences in condition indices [14]. In American red squirrel (*Tamiasciurus hudsonicus* Erxleben, 1777), there were no or very small differences in the body mass between experimentally fed and control populations, although males tended to be heavier in a fed population in winter [16], while urban female eastern chipmunks (*Tamias striatus* L., 1758) were in better body condition than their non-urban conspecifics [17].

Increased year-round food resources in urban conditions results in changes to the onset and duration of breeding seasons, which was observed in numerous animals [18]. A striking example of such is seen in the tawny owl (*Strix aluco* L. 1758), which, in cities starts laying eggs a few weeks earlier even than in non-urban populations, potentially due to, among other reasons, a stable food base [11,19]. Indeed, a case of American red squirrel showed that the fed population had a longer breeding season, with some females having more than one litter [16], which (also thanks to an increased survival) led to a ten-fold increase in the density of the population [20].

The Eurasian red squirrel (*Sciurus vulgaris* L. 1758) has adjusted well to urban habitats [21,22,23,24,25,26,27]. This species is strongly habituated to human presence, and its behavioural flexibility helps it to adjust to these specific conditions [28]. Urban habitats serve as suitable refugia for red squirrels [28,29], who are known to utilise urban structures [30] and move through built-up landscapes [31]. The abundance of red squirrels increases with human population density [29] and can be higher in cities than in rural areas [23,32,33]. Urban red squirrels can change their activity pattern [34], interact more with humans [23,24] and be bolder than their non-urban counterparts [35]. Nevertheless, little is known about the body mass, condition and breeding of typically urban populations of red squirrels. In urban conditions, red squirrels are offered various sources of supplementary food, including the opportunity to gather food from bird feeders, as well as the provision of nuts offered directly by park visitors [22,23,25,36,37,38]. It has been already shown that supplemental food is a key factor that may attract squirrels, and this extra food is most important when natural food is scarce [29,39]. Indeed, in Hamburg, in a supplementarily fed population, red squirrels maintained stable body mass over the course of a year, likely indicating that they were never food-stressed [25,38]. Moreover, individuals whose core areas were located closer to high-energy anthropogenic food sources were heavier, while natural food sources did not affect body mass [25].

The aim of this study was to compare two red squirrel populations inhabiting the same city but two different areas: an urban park and an urban forest, both placed within city districts. Nevertheless, one is expected to be highly affected by human and plentiful supplementary food from park visitors, whereas the effect of human disturbance on the other population should be inconsiderable. Indeed, earlier studies showed that park squirrels lived at higher densities and occupied smaller home ranges. They also changed their behaviour and feeding strategies in response to human presence: they spent more time on the ground, tolerated close contact, and human-delivered nuts made up the bulk of their diet [23,24]. They also changed their activity pattern, making the most of human presence [23,24,34]. In this work, we aimed to compare the body mass and body condition of both sexes and the reproductive activity of females between the two populations. Because food availability affects the ecology of red squirrels [29,31,40,41], we can hypothesise that park squirrels, with access to abundant supplemental food and a rich natural food base, will have higher body mass (and better body condition), which remains more stable over the year, and females will start breeding earlier and prolong their breading season in comparison to the urban forest squirrels.

## 2. Materials and Methods

### 2.1. Study Area

**Figure 1:**
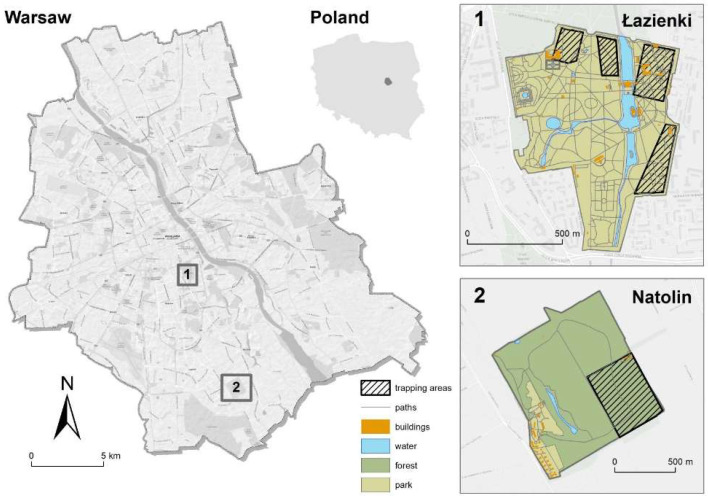
Study areas: 1. Royal Łazienki Museum (an urban park) and 2. Natolin Forest Reserve (an urban forest) in Warsaw, where red squirrels were live-trapped. Approximate locations of trapping areas are shown.

The study was conducted in two sites (Figure 1), both located in Warsaw (52°14′13.37″ N, 21°1′3.11″ E), the capital city of Poland. The city is populated by approximately two million people and is placed in the central part of the country. This region is affected by both the harsh and dry continental climate of Eastern Europe and Asia and the mild oceanic climate of Western Europe. Growing season lasts for about 210 days, and the total precipitation measures 600 mm per year. The mean ambient temperature is from −4 °C in January to 18 °C in July.

The first study site was Royal Łazienki Museum, a park located in the city centre (hereafter ‘urban park’) (Figure 1). This park is very popular among local inhabitants and visitors. It is difficult to estimate how many people visit the park because no entrance ticket is required, and the number of people passing through the gates is not monitored. According to an annual report made by the Warsaw Tourism Organization, the Royal Łazienki Museum park was visited by 3.5 million people in 2018, in 2019, by almost 5 million, and in 2020, by more than 4 million [42]. The urban park covers 76 ha, and it is surrounded by busy streets and built-up areas. The park has more than 90 species of trees and shrubs, both native and foreign species. Deciduous trees are most numerous, e.g., common hornbeam (*Carpinus betulus* L.), common oak (*Quercus robur* L.), common beech (*Fagus sylvatica* L.), as well as common hazel (*Corylus avellana* L.), English walnut (*Juglans regia* L.) and North American walnut (*Juglans nigra* L.). Tree stands can reach more than 150 years old, and numerous old trees provide a rich natural food base for animals [32]. Squirrels here are fed by park visitors every day; some individuals even deliver food for animals on an everyday basis [43]. According to our radio telemetry data and direct observations of tagged squirrels, squirrels took food from humans on average 0.89 times per hour [44]. Most (almost 90%) of the recorded food items eaten by red squirrels in the park were nuts, and most of those (66%) were provided by park visitors [23]. Moreover, there are about 10 feeders located in the park (and the presence of feeders is known to attract squirrels and to be able to increase their abundance [45]), which are stocked with seeds and nuts by park employees during the period September–March. However, feeders stay in the park all year round, and visitors leave food in them for animals throughout the remainder of the year, too.

The second study site was Natolin Forest Reserve, located approximately 10 km from the city centre (hereafter ‘urban forest’) (Figure 1). This land was formerly parkland that extended around the residences of Polish magnates. The spontaneous regeneration of woodland took place after the Second World War, so the reserve area is wholly tree-covered these days. The oldest stands are over 250 years old, and only natural regeneration occurs. Dead and fallen trees are left for natural decomposition. The reserve covers 105 ha, and it has been protected since 1991. Since then, it has been closed to the public. To the west and north of the reserve are built-up areas, whereas on the other sides, it is surrounded by patches of farmland (mostly set-asides), which are successively built-up. Trees are mostly deciduous, e.g., common hornbeam, common oak, ash (*Fraxinus excelsior* L.), elms (*Ulmus* spp.), common hazel and black alder (*Alnus glutinosa* L. (GAERTN.)).

No food base availability assessment was carried out for this study. Nevertheless, tree inventory was previously conducted in 400 m^2^ patches, which were placed exactly in our trapping areas [46]. It was found that the density of trees and shrubs was higher in the urban forest (44/400 m^2^) than in the park (19/400 m^2^); however, trees over 30 years old (which are assumed to be seed-bearing) were more numerous in the urban park than in the forest (40% vs. 11%) [46].

In the past studies, the density of the urban park red squirrels was estimated to be more than 2 individuals/ha [23,32], whereas in the urban forest, it was 0.29 ind./ha [23].

### 2.2. Live-Trapping

The study started in July 2018 and ended in December 2020. Squirrels were live-trapped with 30 traps in the urban park and 40 traps in the urban forest. We used standard wire mesh live traps (51 × 15 × 15 cm) (manufactured by “Jerzyk” Jerzy Chilecki, Białowieża, Poland). The traps were partly covered by dark plastic to provide shelter from rain and snow and were located on the ground or in trees on a wooden platform. Live traps were pre-baited with hazelnuts and English walnuts for seven days, then baited and set for four (in most cases) to nine days. In both areas, we trapped in the same month, during thirteen trapping sessions in total (i.e., in 2018: Jul, Sept, Nov; in 2019: Jan, Mar, May, Jul, Sept; in 2020: Mar, May, Jul, Oct, Dec). The traps were set in the morning (around 6–7 a.m., depending on the time of dawn), checked after 2–4 h and secured for the night in a manner which prevented them from being closed. We flushed every trapped squirrel into a wire mesh handling cone [47] to minimise stress during handling. Each newly trapped squirrel was individually marked with numbered ear-tags 2 × 8 mm (National Tag&Band, Newport, KY, USA). Squirrels were weighed to the nearest 10 g (Pesola spring balance), and the right hind foot, excluding the claw, was measured with tape measure. Body condition was calculated based on residuals of log body mass and foot length [48]. A linear regression model was built using data on 50 adult individuals (log body mass = 2.211 (±0.074) + 0.006 (±0.001) foot length, R^2^ = 0.29, *p* < 0.001). Then, the regression model was used to calculate the residuals of body mass for all individuals. We also defined the sex and reproductive status of females. Females were defined as non-breeding (anoestrous, small vulva, no longitudinal opening), postoestrous and pregnant (swollen vulva with longitudinal opening, enlarged belly during pregnancy) or lactating (large nipples, milk excretion could be stimulated) [49]. We also defined if squirrels were adult or sub-adult. Sub-adult males had small scrotum and abdominal testes, and females had a very small vulva and the nipples were still invisible. Older animals were considered as adult [50].

**Figure 2:**
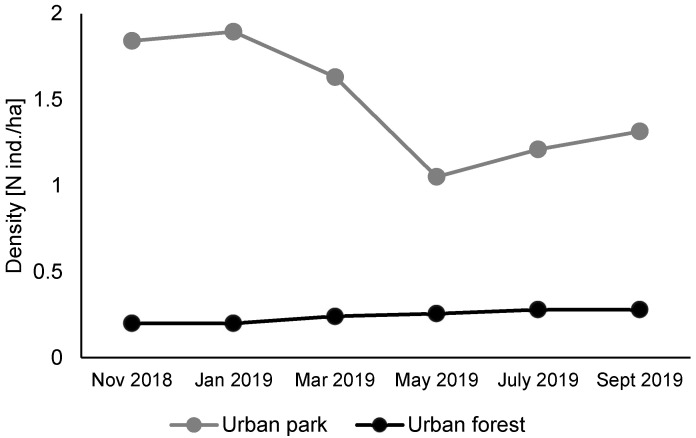
Density of the two studied populations of red squirrels in Warsaw: urban park and urban forest, assessed on the basis of live-trapping and MNA estimates.

We used the minimum number of animals known to be alive (MNA) (e.g., [41,47,51,52]) from the trapping during period October 2018–September 2019. We estimated squirrel density using edge-correction using the average female range radius in study sites [41]. According to a previous study [23], the average radius for females in the urban park was 75 m, while in the urban forest, it was 135 m. MNA estimates assessed during the first year of this study confirmed this difference in density between the two populations: the value obtained for the urban park squirrels ranged from 1.05 to 1.89 ind./ha and from 0.2 to 0.28 ind./ha for forest squirrels (Figure 2).

Access to the Natolin Forest Reserve and red squirrel capture was allowed with the permission issued by the General and Regional Directorates for Environmental Protection. The trapping and handling of squirrels complied with current laws on animal research in Poland and were carried out with a permit from the Local Ethical Committee (WAW2/072/2018).

### 2.3. Statistical Analysis

We compared the sex proportion and age proportion of all individuals across both sites (urban park vs. urban forest) with the Chi-squared tests. We also analysed differences in the body mass and body condition of adult individuals and the reproductive status of adult females between sites (including other factors). We used three models: two linear mixed models (for body mass and body condition) and one generalised linear mixed binary model (logit regression) for reproductive status, where we included all adult females (including recaptures). In the first LMM, the dependent variable was body mass. In this model, we tested the effect of foot length (FOOT), sex (SEX), site (SITE), season (SEASON) and the interaction of site with other two variables: SITE*SEX and SITE*SEASON. SITE was a grouping variable of the two study sites: urban park (Łazienki) and urban forest (Natolin). SEASON was a grouping variable of four astronomical seasons: spring (1 March–31 May), summer (1 June–31 August), autumn (1 September–30 November) and winter (1 December–31 February). In the second LMM, the dependent variable was body condition, and a similar set of variables apart from foot length (FOOT) and interactions between variables was included. In the third model, the reproductive status of adult females was analysed. In this model, all breeding female squirrels (i.e., postoestrous and pregnant or lactating) were marked as 1, and all non-breeding squirrels were marked as 0. In this model, the independent variables were: site (SITE), season (SEASON), body condition (CONDITION) and interaction of site and season (SITE*SEASON). We did not include the body mass as it was highly correlated with the body condition (Pearson’s r = 0.849). The IDs of squirrels were set as a random effect in both models to account for the repeated sampling of individual animals. Restricted maximum likelihood (REML) was used to estimate the parameters in the best model obtained. Model selection was based on *p*-values in stepwise backward selection [53]. Groups within variables included in the best model were compared with the LSD test.

All statistical analyses were performed with SPSS software (version 26.0, IBM, Armonk, NY, USA).

## 3. Results

In total, 36 squirrels (19♀, 17♂) were trapped in the forest, while 106 squirrels (48♀, 58♂) were trapped in the park. In the forest, squirrels were trapped in total 129 times (Mean = 3.58, Min = 1, Max = 11), and in the park, 266 times (Mean = 2.44, Min = 1, Max = 12).

**Figure 3:**
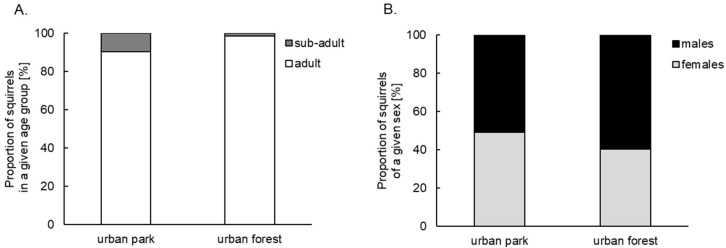
Proportion of red squirrels of given (**A**) age and (**B**) sex groups in the urban park and urban forest.

The share of caught sub-adults in the forest did not exceed 2% of all caught individuals, while in the park, this age group constituted almost 10% (Figure 3A). The proportions were significantly different (χ^2^ = 8.92, *p* = 0.003). The proportions of males and females of all individuals did not statistically differ between the areas (χ^2^ = 2.79, *p* = 0.095) (Figure 3B).

**Figure 4:**
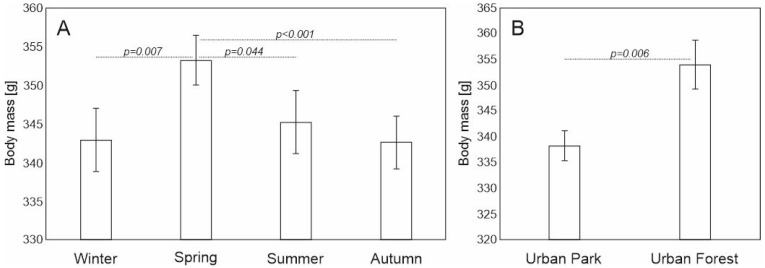
Mean (±SE) body mass of adult red squirrels with regard to (**A**) season and (**B**) study site, and pairwise comparison with Least Significant Difference test in LMM (significant differences presented above the bars). Please note that Y axis does not start from 0 value.

Adult red squirrels in the forest had higher body masses (Mean = 355 g, ±SD = 29) than those in the park (Mean = 337.2 g, ±SD = 33.2) (Figure 4B). The analysis of body mass showed that the model, after selection, contained three explanatory variables: FOOT, SITE and SEASON (Table S1). Regarding seasonal differences in the body mass, only spring differed significantly from other seasons (summer *p* = 0.044, autumn *p* < 0.001, winter *p* = 0.007) (Figure 4A).

**Figure 5:**
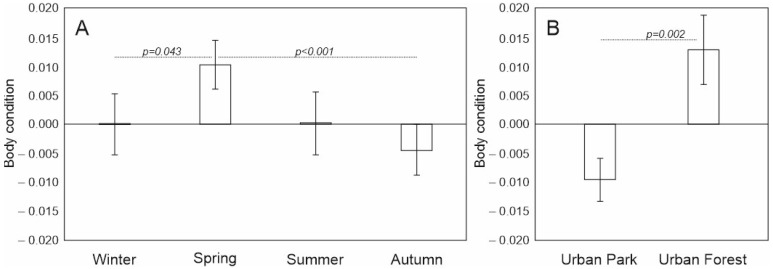
Mean (±SE) body condition of adult red squirrels with regard to (**A**) season and (**B**) study site and pairwise comparison with Least Significant Difference test in LMM (significant differences presented above the bars).

The squirrels in the urban forest had better body conditions than those in the urban park (Mean = 6.58, ±SD = 0.53 and Mean = 6.21, ±SD = 0.63, respectively), and the difference was statistically significant (*p* = 0.002) (Figure 5B). Furthermore, body condition was higher in spring compared to autumn and winter (*p* = 0.001, and *p* = 0.043, respectively) (Figure 5A). Body condition did not differ with regard to sex or subgroups in the interactions and was excluded during model selection (Table S2).

**Figure 6:**
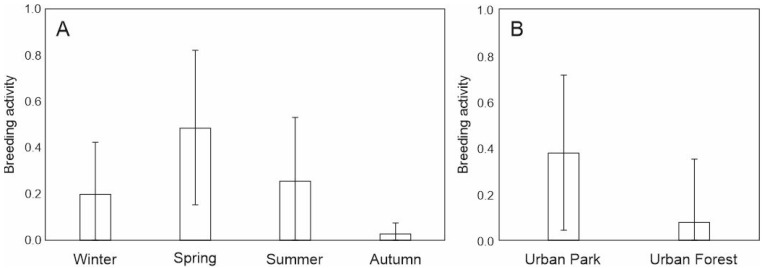
Frequency (±SE) of breeding (i.e., postoestrous and pregnant, lactating) adult female red squirrels with regard to (**A**) season and (**B**) study site. No statistically significant differences in pairwise comparisons using the Least Significant Difference test.

During the whole study period, the proportion of sexually active adult females in the urban forest was lower than in the urban park (23% and 35%, respectively). The best fit model contained all variables: study site, season and condition of animals (Table S3). The probability of being sexually active (i.e., breeding) increased with the body condition of females (β = 9.38, SE = 4.37, *p* = 0.033). The highest frequency of sexually active squirrels was observed in spring and summer, but sexually active individuals were also found in winter (Figure 6A). A higher frequency of sexually active adult squirrels was observed in an urban park (Figure 6B). No statistically significant differences were stated in pairwise comparison. The minimum body mass of breeding females was 350 g in the forest and 300 g in the park.

## 4. Discussion

As we predicted, the two populations of squirrels, one living in a busy urban park, and the other from an urban forest, differed in terms of body mass, body condition and breeding activity.

First, forest squirrels were heavier and had better body conditions than those from the park. Differences in the body masses between squirrels of the same population can be caused by variation in their size or in the amount of fat under the skin [54]. Body condition and habitat quality are strongly linked [54], so we may have expected that the year-round supplementary feeding of park squirrels [22,23,24,34] would result in increased body condition [17]. However, surprisingly, forest squirrels were generally in better body condition than park squirrels, who had access to year-round supplemental feeding. Optimal body mass and a layer of fat is crucial for squirrels to ensure thermal insulation and energy during periods of food shortage [55]. Habitat and population density may affect body mass, too. In general, squirrels inhabiting deciduous habitats had lower body mass than squirrels living in conifer habitats [54]. In our case, both study sites were deciduous; however, in the urban park, population density was much higher than in the urban forest. It has been reported that squirrels of high social rank, both males and females, weigh more than squirrels of low rank, and body mass may be positively correlated with boldness [56] and aggressiveness [54,57]. In high-density populations, squirrels may be less aggressive towards conspecifics [58]. It may thus be argued that in this high-density population, more subdominant squirrels with lower body mass survived. In the forest, in turn, access to large nuts (in our case hazelnuts, hornbeam nuts, and acorns) allowed the population to reach a higher body mass, which is crucial for settlement success and local survival [40]. Our results are to some extent in line with other studies in which body mass and/or condition were not driven by supplementary feeding [39,59,60]. According to Magris and Gurnell [39], supplementary food can affect squirrel density and population biomass (which is the case of park squirrels in our study) but does not affect individual body mass and condition.

In general, the body mass of red squirrels may change seasonally, according to food availability, weather and reproductive status [54,61]. In deciduous habitats, body mass is typically highest in autumn [55,62], when squirrels feed mainly on high-energy tree seeds and accumulate fat for the winter period [54,62]. On the other hand, no autumn or winter increase in body weight was observed in coniferous habitat, which was explained by more predictable food supplies [61]. In winter, food availability remains high, but due to high thermoregulation costs, fat reserves may become depleted [55]. Next, during spring, fat reserves become further depleted because of the high energy cost of reproduction and increased activity [55]. Therefore, squirrels with higher body mass and larger reserves of fat are better equipped to cope with stressful conditions, which in turn increases their chances of survival [40,49,54]. In our study, a slight seasonal variation in the body mass of squirrels was found, but surprisingly, they were heaviest in spring, with no differences between other seasons. This pattern of changes in the body mass stands in contrast to published findings (see above). There was no contrast between summer–autumn–winter activity in our study. Our forest squirrels were seen to typically reduce their winter activity, which is a response to high thermoregulatory costs [36,63]. This may help them to retain high fat resources regardless of changing environmental conditions. Additionally, recent mild winters [64] together with an influence of an urban island heat effect, reaching as far as the outer districts of Warsaw [65], may be facilitative. As shown in a previous study, forest squirrels occupied relatively small home ranges [23], which suggested rich natural food supply [61]. In contrast, park squirrels were more active, assumingly trying to adjust activity to the reduced presence of park visitors in winter [34], which might help them to retain fat resources. Our park is located in the central part of the city, where the temperatures were the highest [65], so squirrels could spend more time outside their dreys. On the other hand, in spring, which normally is a time of low food availability [62,66], park visitors (i.e., food providers) became abundant, which assumingly helped squirrels to restore fat reserves quickly. Low variation in the body mass during other seasons in the case of park squirrels is in line with findings from Hamburg, where in a fed population, individual body mass was stable [25], assumingly due to high and stable food energy availability [67].

For females, body mass is especially important, as a fat layer provides a valuable energy store during lactation and nursing young [55]. The reproductive success of females increases with their body mass and body condition [54,55,68]—heavier squirrels grow older and produce more offspring per litter and more litters in their lifetime [40,48,49,54,55]. Additionally, in our study, females in better body condition were more likely to breed. What is interesting is that although females in the park had generally worse body condition, the overall proportion of sexually active females was higher compared to the forest squirrels. A minimum body mass is required to enter oestrus [39,54], and this value was lower for park than forest squirrels. Squirrels in the park are fed by people during all seasons [22,23]; thus, probably, squirrels do not need such large fat reserves to commence breeding and produce successfully. Our study only specified very simple breeding parameters and did not measure the breeding success of females. Nevertheless, more sub-adult individuals were trapped in the park than in the forest, which suggested a higher reproduction rate. This is consistent with a study on fox squirrels, which were more often reproductively active and presented higher juvenile survival and juvenile/adult ratios in the urban population than the rural one [69]. Additionally, juvenile and sub-adult squirrels appeared more frequently in an experimentally supplementary fed American red squirrel population as a result of a longer breeding period [16].

Red squirrels are seasonal breeders; females have one or two litters during a year: first in February–March and second in June–July [51,52,70,71]. Seed availability can affect, among other things, the length of the breeding season and the number of adults which produce two litters [68]. The frequency of breeding females was, regardless the study area, highest in spring, but squirrels also bred in winter. This early reproduction may have been possible thanks to good feeding conditions and higher winter temperatures in the city [72]. Females giving birth early in the year would have sufficient time to produce a second litter [68]. Additionally, by breeding early, females may be able to enhance the chances of their offspring to settle in an optimal home range as juveniles from early litters can find more vacant areas [59]. As shown by earlier studies, and in line with our findings, the availability of supplemental food had no significant effect on the length of breeding season [59], and early breeding (spring litters) was not affected by food availability [39].

## 5. Conclusions

As was reviewed by Boutin [73], individuals receiving supplemental food usually had higher body mass and advanced breeding relative to those in control areas. In our study, urban park squirrels that received supplemental feeding had lower body mass (and worse body condition) than those inhabiting the urban forest, and there was no difference in breeding time. This difference in the body mass/condition may be explained by high intraspecific competition in a very abundant squirrel population. On the other hand, stable food conditions (i.e., year-round supplemental feeding) combined with less demanding weather conditions typical for the city centre might mean that accumulating fat is not crucial for survival and breeding. Abundant supplemental food may also result in a higher share of breeding females. These differences between the two populations complete our earlier findings on how far urban park and urban forest squirrels differed in certain aspects of their ecology (i.e., space use, food choice, activity patterns and interactions with people) and proved the high plasticity of squirrels inhabiting urban landscapes.

## Data Availability

The data presented in this study are available on request from the corresponding author.

## Supplementary Materials

The following supporting information can be downloaded at: https://www.mdpi.com/article/10.3390/ani12233246/s1, Table S1: Effects of a site (SITE), season (SEASON) and foot length (FOOT) on body mass of squirrels in a general linear mixed model; Table S2: Effects of site (SITE) and season (SEASON) on body condition of squirrels in a general linear mixed model; Table S3: Effects of site (SITE), season (SEASON) and body condition (CONDITION) on sexual activity of red squirrels in generalised linear binary model.
