# Supplementary material for: Body Condition and Breeding of Urban Red Squirrels: Comparison of Two Populations Affected by Different Levels of Urbanization
Source: Animals (Basel). 2022 Nov 23;12(23):3246. doi: 10.3390/ani12233246 (PMC9736854; doi:10.3390/ani12233246)
Supplement: Supplementary file 1 [file animals-12-03246-s001.zip › Supplementary material.pdf]

Supplementary material 1

Table S1. Effects of a site (SITE), season (SEASON), and foot length (FOOT) on body mass of squirrels in a general linear mixed model, * reference category.

**Source**	***B***	***SE***	***t***	***p***	***Lower CI***	***Upper CI***
Intercept	261.18	27.76	9.41	<0.001*	206.57	315.78
SITE (Łazienki)	-15,68	5.60	-2.80	0.006*	-26.77	-4.58
SITE (Natolin)	0*					
SEASON (winter)	-10.28	3.80	-2.71	0.007*	-17.76	-2.80
SEASON (autumn)	-10.57	3.17	-3.33	<0.001*	-16,81	-4.33
SEASON (summer)	-7.98	3,95	-2.02	0.044*	-15.75	-0.22
SEASON (spring)	0*					
FOOT	1.84	0.51	3,64	<0.001*	0.85	2.84

Table S2. Effects of site (SITE) and season (SEASON) on body condition of squirrels in a general linear mixed model, * - reference category.

**Source**	***B***	***SE***	***t***	***p***	***Lower CI***	***Upper CI***
Intercept	0.02	0.01	3.35	<0.001*	0.01	0.04
SITE (Łazienki)	-0,02	0.01	-3.17	0.002*	-0.03	-0.01
SITE (Natolin)	0*					
SEASON (winter)	-0.01	0.01	-2.02	0.043*	-0.02	0.00
SEASON (autumn)	-0.02	0.00	-3.45	<0.001*	-0.02	-0.01
SEASON (summer)	-0.01	0.01	-1.91	0.057	-0.02	0.00
SEASON (spring)	0*					

Table S3. Effects of site (SITE), season (SEASON) and body condition (CONDITION) on sexual activity of red squirrels in generalized linear binary model, * - interaction.

**Source**	***B***	***SE***	***t***	***p***	***Exp (B)***	***Lower CI***	***Upper CI***
Intercept	0.27	1.47	-0.19	0.853	1.31	0.73	23.42
SITE (Natolin)	-1,03	0.46	-2.24	0.026*	0.36	0.04	0.30
SITE (Łazienki)	0*						
SEASON (winter)	-0.49	0.38	-1.30	0.195	0.61	0.29	1.29
SEASON (autumn)	-2.75	0.46	-5.99	<0.001*	0.06	0.03	0.16
SEASON (summer)	-2.30	0.55	-4.14	<0.001*	0.10	0.03	0.30
SEASON (spring)	0*						
CONDITION	9.38	4.37	2,15	0.033*	$119*10^{2}$	2.18	$646*10^{5}$
